# A comprehensive analysis identifies and validates NPC1 as a potential biomarker for prognosis in HCC

**DOI:** 10.3389/fgene.2025.1588583

**Published:** 2025-08-13

**Authors:** Xingjun Lu, Tianyao Gou, Xuxing He, Xia Chen, Daqing Yang, Xiaozhen Peng

**Affiliations:** ^1^ College of Laboratory Medicine, Hunan University of Medicine, Huaihua, China; ^2^ Xiangya Second Hospital, Central South University, Changsha, China; ^3^ Clinical Medicine Department, Xinjiang Medical University, Urumqi, China; ^4^ Department of Neurology, The First Affiliated Hospital of Hunan University of Medicine, Huaihua, China; ^5^ Hunan Provincial Key Laboratory for Synthetic Biology of Traditional Chinese Medicine, Hunan University of Medicine, Huaihua, China

**Keywords:** NPC1, prognosis, biomarker, HCC, therapeutic target

## Abstract

Niemann-Pick type C1 protein (NPC1), a key regulator of intracellular cholesterol transport and a transmembrane protein, has been implicated in carcinogenesis, particularly in hepatocellular carcinoma (HCC). Despite the noted association, the specific role of NPC1 in HCC remains underexplored. In this study, we conducted a comprehensive analysis of NPC1 expression across diverse gene expression databases to elucidate its prognostic significance and functional interactions. Utilizing LinkedOmics for co-expression network analysis and KEGG for functional enrichment, we identified a set of genes co-expressed with NPC1 and its associated biological pathways. Our findings demonstrate that NPC1 is frequently upregulated and amplified in HCC tumor tissues, with higher expression levels significantly associated with reduced overall survival (OS), progression-free survival (RFS), and disease-free survival (DFS). Functional enrichment analysis of the top 50 positively correlated genes with NPC1 highlighted significant enrichment in pathways related to organelle fission, nuclear division, chromosomal region spindle formation, and centrosome function, suggesting a role for NPC1 in DNA replication processes. These findings establish a correlation between NPC1 expression and HCC prognosis, laying the groundwork for future studies to explore the therapeutic potential of NPC1 inhibition in HCC.

## 1 Introduction

HCC has emerged as a formidable adversary in the global oncological landscape, being one of the most common cancers and a top cause of cancer deaths worldwide (Akinyemiju et al., 2017; Yang et al., 2019). The etiology of HCC is multifaceted, with chronic infections by hepatitis B or C viruses, alcohol-induced liver diseases, and non-alcoholic steatohepatitis being the primary risk factors implicated in its development (Makarova-Rusher et al., 2016; Zhang et al., 2004). The molecular underpinnings of HCC initiation and progression remain elusive, presenting a significant challenge to the development of effective therapeutic interventions. Consequently, there is an urgent need to unravel the molecular mechanisms at play in HCC pathogenesis to facilitate the discovery of novel therapeutic strategies that could improve patient survival outcomes.

The cellular metabolism of cholesterol, a critical biomolecule, involves the internalization of low-density lipoproteins (LDL) rich in cholesterol through endocytic pathways. Within the lysosome, cholesterol is metabolized, and the protein NPC1 plays a crucial role in transporting free cholesterol from the lysosome to the cytosol (Pfeffer, 2019; Davies et al., 2000; Goldstein and Brown, 2015). NPC1, a transmembrane protein located at the lysosomal limiting membrane, is a key regulator of cellular cholesterol uptake from extracellular sources (Skorda et al., 2023). Loss-of-function mutations in NPC1 lead to the development of Niemann-Pick type C disease, characterized by the pathological accumulation of cholesterol within lysosomes, which disrupts lysosomal trafficking and function (Li et al., 2016; Kennedy et al., 2014; Geberhiwot et al., 2018). Recent research has highlighted the significant role of lysosomes in cancer biology, with exocytosed lysosomal hydrolases capable of promoting extracellular matrix degradation and cancer cell invasion (Rafn et al., 2012; Machado et al., 2021; Kallunki et al., 2013). This places NPC1 at the forefront as a potential therapeutic target in cholesterol trafficking and a candidate for cancer therapy. However, the precise role and the underlying mechanisms of NPC1 in HCC progression are yet to be fully delineated.

We analyzed NPC1 expression across multiple tumor types, with a particular focus on HCC. We investigated its correlation with prognostic indicators and clinical features within the context of HCC. This study suggested that NPC1 could be a valuable biomarker for identifying, predicting outcomes, and assessment of treatment response in HCC patients, offering new avenues for clinical management and therapeutic intervention.

## 2 Methods

### 2.1 Quantitative analysis of NPC1 expression across multiple datasets

To ascertain the expression profile of NPC1 in HCC, we systematically retrieved gene expression data from two comprehensive biorepositories: The International Cancer Genome Consortium (ICGC) and the Cancer Genome Atlas (TCGA, normalNum = 50, tumorNum = 374). The ICGC and TCGA datasets were reconciled using quantile normalization and batch effect removal using svaseq as previously described (Xiong et al., 2022).

Data extraction was facilitated by employing R software, a robust statistical programming environment. In order to substantiate the transcriptional levels of NPC1 within HCC, we further incorporated samples from the Genotype-Tissue Expression (GTEx) project and TCGA, leveraging the analytical capabilities of the Gene Expression Profiling Interactive Analysis (GEPIA), Oncomine, and UALCAN databases. Furthermore, we scrutinized NPC1 expression patterns across a series of Gene Expression Omnibus (GEO) datasets, specifically GSE45436 (normalNum = 41, tumorNum = 93), GSE76427 (normalNum = 52, tumorNum = 115), GSE64041 (normalNum = 5, tumorNum = 60), GSE10140_GSE10141 (normalNum = 82, tumorNum = 80), and GSE102079 (normalNum = 91, tumorNum = 152).

To bridge the gap between gene expression and protein functionality, we employed immunohistochemistry (IHC) data from the Human Protein Atlas (HPA) database to evaluate NPC1 protein expression levels in HCC. Concurrently, we harnessed the Clinical Proteomic Tumor Analysis Consortium (CPTAC) proteomics database to ascertain the protein levels of NPC1, thereby providing a multifaceted view of NPC1 expression dynamics in HCC.

### 2.2 Survival analysis

To evaluate the prognostic value of NPC1 in HCC, a survival analysis was carried out with data from the TCGA database. The prognostic importance of NPC1 expression levels in HCC patients was evaluated using the Kaplan-Meier plotter. This method enables the generation of survival curves and the estimation of hazard ratios (HR), which are crucial for assessing the impact of NPC1 on patient survival. To ensure the robustness of our findings, we used R software (version 3.5.2) to validate the prognostic relevance of NPC1 in independent datasets, including the ICGC database and the GSE76427 dataset. The relationship between NPC1 expression and survival outcomes in HCC was comprehensively analyzed using HR with 95% confidence intervals (CI) and log-rank p-values.

### 2.3 Association of NPC1 expression with clinical characteristics in HCC

We examined NPC1 expression in relation to clinical characteristics in HCC patients. The analysis was performed using R software, a comprehensive statistical programming environment, which allowed us to analyze the association between NPC1 expression and clinical parameters. To validate our findings, we utilized the UALCAN database, an online resource that facilitates the analysis of cancer gene expression in relation to clinical features. We employed the t-test or ANOVA to assess the statistical significance of differences in NPC1 expression levels across different groups, based on clinical characteristics.

### 2.4 LinkedOmics database analysis

The LinkedOmics database, an invaluable resource for multi-dimensional analysis of the TCGA cancer data, was utilized to explore the co-expression patterns of NPC1. We employed Pearson’s correlation coefficient to statistically analyze the co-expression of NPC1 with other genes within the TCGA dataset. The results were visualized using various graphical representations, including heat maps, scatter plots, and volcano plots, which facilitated the identification of genes with significant correlation to NPC1. To further elucidate the biological significance of NPC1, we performed Gene Ontology biological process (GO_BP) enrichment, KEGG (Kyoto Encyclopedia of Genes and Genomes) pathway analysis, and targeted enrichment analysis of kinases, microRNAs (miRNAs), and transcription factors (TFs) using the Gene Set Enrichment Analysis (GSEA) within the LinkedOmics functional modules. The significance criteria required a false discovery rate (FDR) of less than 0.01 for gene expression and a p-value of less than 0.05 for gene correlation.

### 2.5 Gene set enrichment analysis

GSEA is a computational tool developed to evaluate if a predetermined set of genes exhibits significant expression differences between two biological states or phenotypes.

In this study, we applied GSEA to identify genes that are differentially expressed based on their correlation with NPC1 expression levels. This analysis aimed to reveal significant survival differences between groups with high and low NPC1 expression. A total of 1,000 permutations were conducted for each GSEA to ensure robustness of the results. Phenotypes were labeled according to NPC1 expression levels, and the pathways enriched in each phenotype were classified using p-values and normalized enrichment scores (NES).

### 2.6 Statistical analysis

All statistical analyses involving data from TCGA were performed using R version 3.6.1. The differential expression of NPC1 across the ICGC, TCGA, and CPTAC cohorts was assessed using the limma and vioplot packages. Survival analysis for samples from the ICGC was conducted using the Kaplan-Meier Plotter. Additionally, the correlation of NPC1 gene expression with clinical outcomes was evaluated in the TIMER and GEPIA databases employing Spearman’s correlation analysis.

## 3 Results

### 3.1 Differential expression of NPC1 in tumor and normal tissues

**FIGURE 1:**
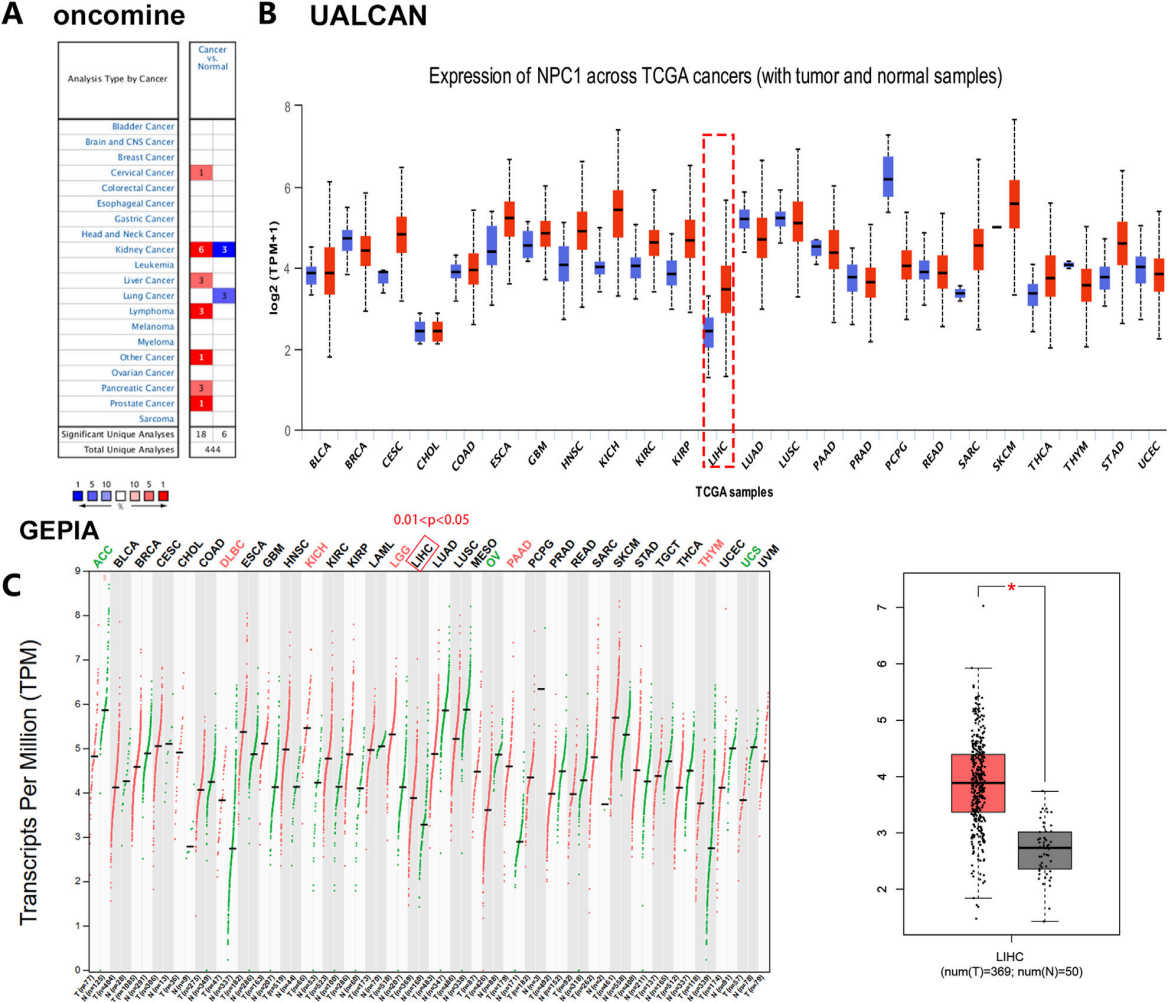
Comparative analysis of NPC1 expression in tumor and normal tissues. The differential NPC1 expression in cancers versus adjacent normal tissues across various cancer types, as retrieved from the TCGA database and analyzed using Oncomine **(A)**, UALCAN **(B)**, and GEPIA database **(C)**. In the left panel of Figure 1C, NPC1 expression in normal versus tumor tissues across various cancer types. In the right panel of Figure 1C, NPC1 expression in normal vs tumor (LIHC) tissues. The statistical significance of the differences in NPC1 expression is denoted by asterisks: *P < 0.05, **P < 0.01, ***P < 0.001. The full form of the abbreviation: BRCA: Breast cancer (BRCA), Cervical squamous cell carcinoma (CESC),Cholangiocarcinoma (CHOL), Colon adenocarcinoma (COAD), Diffuse large B-cell lymphoma (DLBC), Esophageal carcinoma (ESCA), Glioblastoma multiforme (GBM), Head and neck squamous cell carcinoma (HNSC), Kidney chromophobe (KICH), Kidney renal clear cell carcinoma (KIRC), Kidney renal papillary cell carcinoma (KIRP), Acute myeloid leukemia (LAML), Lower grade glioma (LGG), Liver hepatocellular carcinoma (LIHC), Lung adenocarcinoma (LUAD), Lung squamous cell carcinoma (LUSC), Mesothelioma (MESO), Ovarian cancer (OV), Pancreatic adenocarcinoma (PAAD), Pheochromocytoma and paraganglioma (PCPG), Prostate adenocarcinoma (PRAD), Rectum adenocarcinoma (READ), Sarcoma (SARC), Skin cancer (SKCM), Testicular germ cell tumor (TGCT), Thyroid carcinoma (THCA), Thymoma (THYM), Stomach adenocarcinoma (STAD), Uterine corpus endometrial carcinoma (UCEC), Ocular melanomas (UVM).

Our investigation into the expression patterns of NPC1 across a spectrum of normal and cancerous tissues revealed significant findings. Utilizing the Oncomine database, we observed a pronounced upregulation of NPC1 in the majority of cancer types examined, including cervical, kidney, liver, lymphoma, pancreatic, and prostate cancers (Figure 1A). This initial observation suggests a potential role for NPC1 in oncogenic processes.

Expanding our analysis with the UALCAN database, we identified a significant overexpression of NPC1 in 17 distinct cancer types. In contrast, NPC1 expression was notably lower in six cancer types (Figure 1B). These contrasting patterns of expression underscore the heterogeneity of NPC1’s role in different cancer types.

To further validate the findings, we conducted an analysis of NPC1 expression in liver hepatocellular carcinoma (LIHC) within the TIMER database using the GEPIA platform. This analysis confirmed the significant upregulation of NPC1 in LIHC, aligning with our previous observations (Figure 1C). The consistent upregulation of NPC1 in LIHC across different databases strengthens the evidence for its potential involvement in hepatocellular carcinoma.

In summary, our results demonstrated a complex pattern of NPC1 expression in cancer, with significant upregulation in certain cancer types and downregulation in others. These findings warrant further investigation into the functional implications of NPC1 in tumorigenesis and its potential role as a treatment target or biomarker.

### 3.2 NPC1 expression and its prognostic implications in liver cancer

**FIGURE 2:**
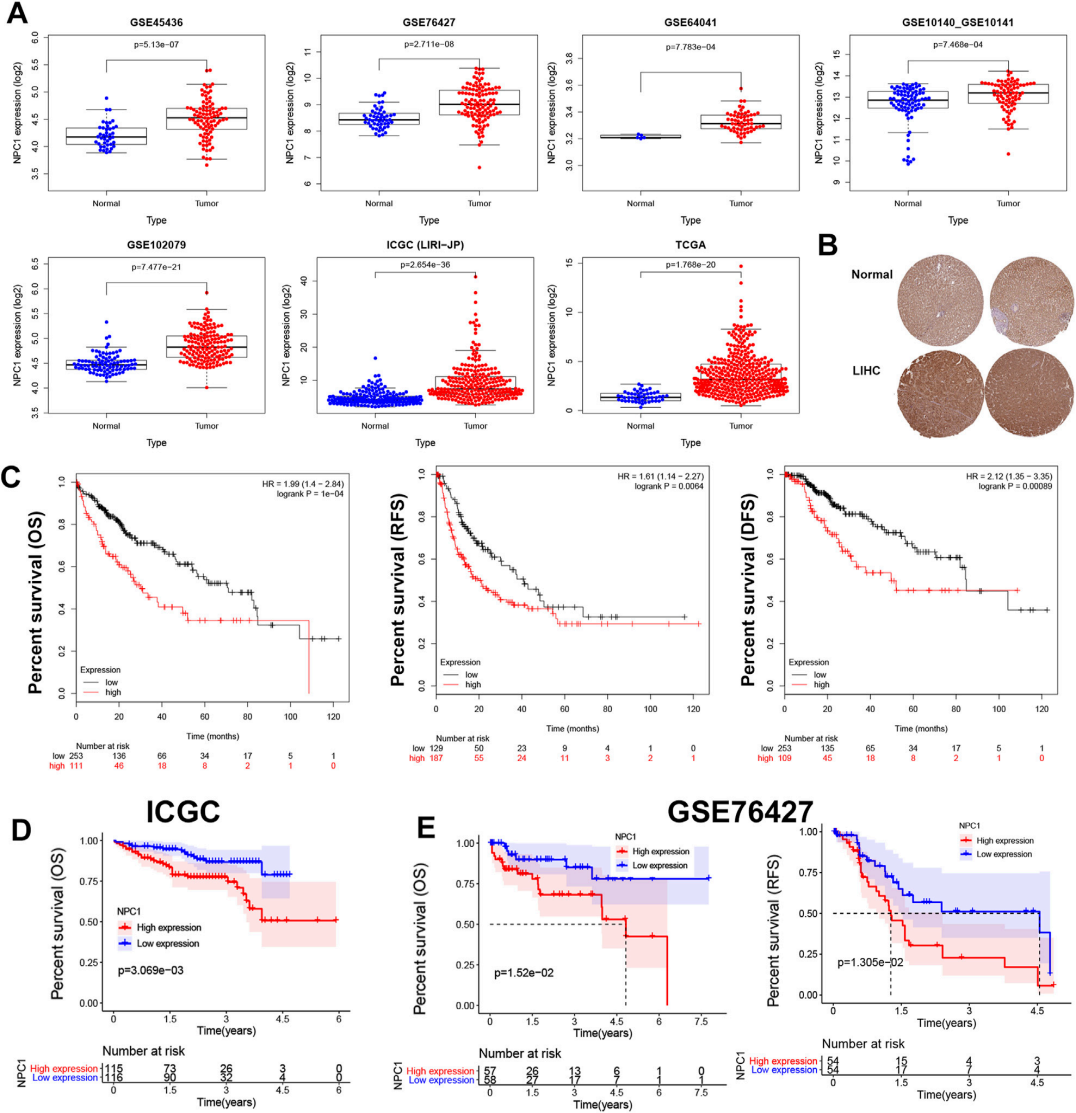
NPC1 expression and prognostic relevance in HCC. The expression levels of NPC1 in liver tumor and normal tissues **(A)**, NPC1 protein expression as per the HPA database **(B)**, and the correlation between NPC1 expression and OS and DFS in HCC using Kaplan-Meier survival curve analysis **(C)**. Additional correlations between NPC1 and OS from the ICGC database were shown in **(D)**, and the correlation between NPC1 and OS and RFS from the GSE76427 database was displayed in **(E)**.

We investigated NPC1 mRNA expression in liver tumor tissue compared to normal tissue across seven datasets: GSE45436, GSE76427, GSE64041, GSE10140, GSE10141, GSE102079, ICGC (LIRI-JP), and TCGA (Figure 2A). Consistent with these molecular findings, Human Protein Atlas (HPA) data presented in Figure 2B revealed significantly higher staining intensity for NPC1 in tumor tissue compared to normal tissue, indicating elevated NPC1 protein expression. This conclusion was further corroborated by the examination of mRNA and protein of NPC1 expression, which demonstrated markedly higher levels in Liver Hepatocellular Carcinoma (LIHC) tumor tissues relative to normal tissues.

Univariate Cox regression and log-rank tests were used to examine the prognostic role of NPC1 in liver cancer. According to the Kaplan-Meier Plotter analysis, increased levels of NPC1 expression were significantly correlated with lower OS [P = 1e-04, HR = 1.99 (1.4–2.84)], RFS [P = 0.0064, HR = 1.61 (1.14–2.27)], and disease-free survival (DFS) [P = 0.00089, HR = 2.12 (1.35–3.35)] in patients with liver cancer (Figure 2C). Conversely, low NPC1 expression was linked to longer OS (p = 3.069e-03) according to the ICGC database (Figure 2D), and longer OS (p = 1.52e-02) and RFS (p = 1.305e-02) as per the GSE76427 database (Figure 2E).

Furthermore, we extended to other cancer types using the Kaplan-Meier plotter database, which showed that high levels of NPC1 expression were linked to poorer OS in patients with Bladder carcinoma, Cervical squamous cell carcinoma, Esophageal carcinoma, Kidney carcinoma, Ovarian cancer, lung carcinoma, Hepatocellular carcinoma,and Pancreatic ductal adenocarcinoma (Supplementary Figure S1). These findings underscore the prognostic significance of NPC1 expression across a range of cancers, including liver, breast, gastric, pancreatic ductal, and lung cancers.

Our results highlighted the prognostic relevance of NPC1 expression in liver cancer and suggested a possible function in various cancer types. The consistent connection between increased NPC1 expression and poor survival outcomes warrants further study into NPC1’s role in tumor progression.

### 3.3 Analysis of clinical information on NPC1 in HCC

**FIGURE 3:**
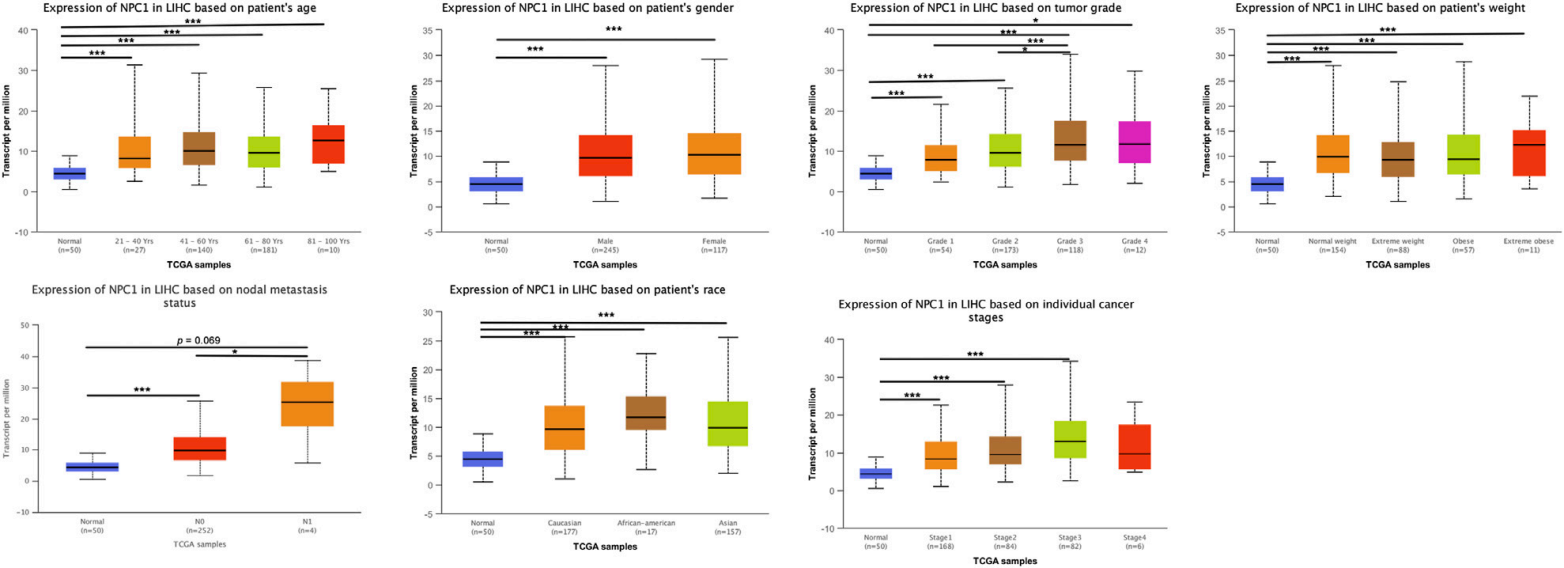
NPC1 mRNA expression in LIHC patients stratified by clinical characteristics. The mRNA expression of NPC1 in LIHC patients based on various clinical characteristics, as analyzed by UALCAN. The t-test was used to assess the significance of differences in gene expression levels between groups, with indicating *p < 0.05, **p < 0.01, and ***p < 0.001.

Subsequently, we undertook a systematic investigation to elucidate the connection between NPC1 expression levels and various clinical parameters in HCC. We examined pathological samples from our patient cohort, and the clinicopathological characteristics are delineated in Figure 3. The HCC patients were stratified into subgroups based on multiple clinical indices, including age, gender, tumor grade, weight, nodal metastasis status, race, and cancer stage.

We also found a statistically significant difference in NPC1 expression between low-grade (Grade 1) and high-grade (Grade 3) cancer groups. This trend was also observed when comparing intermediate-grade (Grade 2) to high-grade (Grade 3) groups, with Grade 3 tumors exhibiting markedly elevated NPC1 levels (Figure 3). This suggests a potential link between NPC1 expression and tumor aggressiveness.

Furthermore, when comparing NPC1 expression levels in relation to nodal metastasis status, we observed an increase in NPC1 expression in the N0 group (no nodal metastasis), which was statistically significant (Figure 3). This finding implies that elevated NPC1 expression may be associated with a reduced likelihood of nodal metastasis, a factor that is typically indicative of better prognosis in cancer patients.

Although NPC1 expression levels did not demonstrate significant differences across age, gender, weight, race, and cancer stage groups, a trend towards higher NPC1 expression was noted in these patient groups. These observations suggest that elevated NPC1 expression may be correlated with adverse clinical features and outcomes.

In aggregate, we found that increased NPC1 expression correlated with increased tumor grade and potentially linked to the metastatic potential of HCC. These discoveries highlighted NPC1 could serve as a prognostic biomarker and called for further investigation into its role in HCC progression and metastasis.

### 3.4 NPC1 as an independent prognostic factor in hepatocellular carcinoma

**FIGURE 4:**
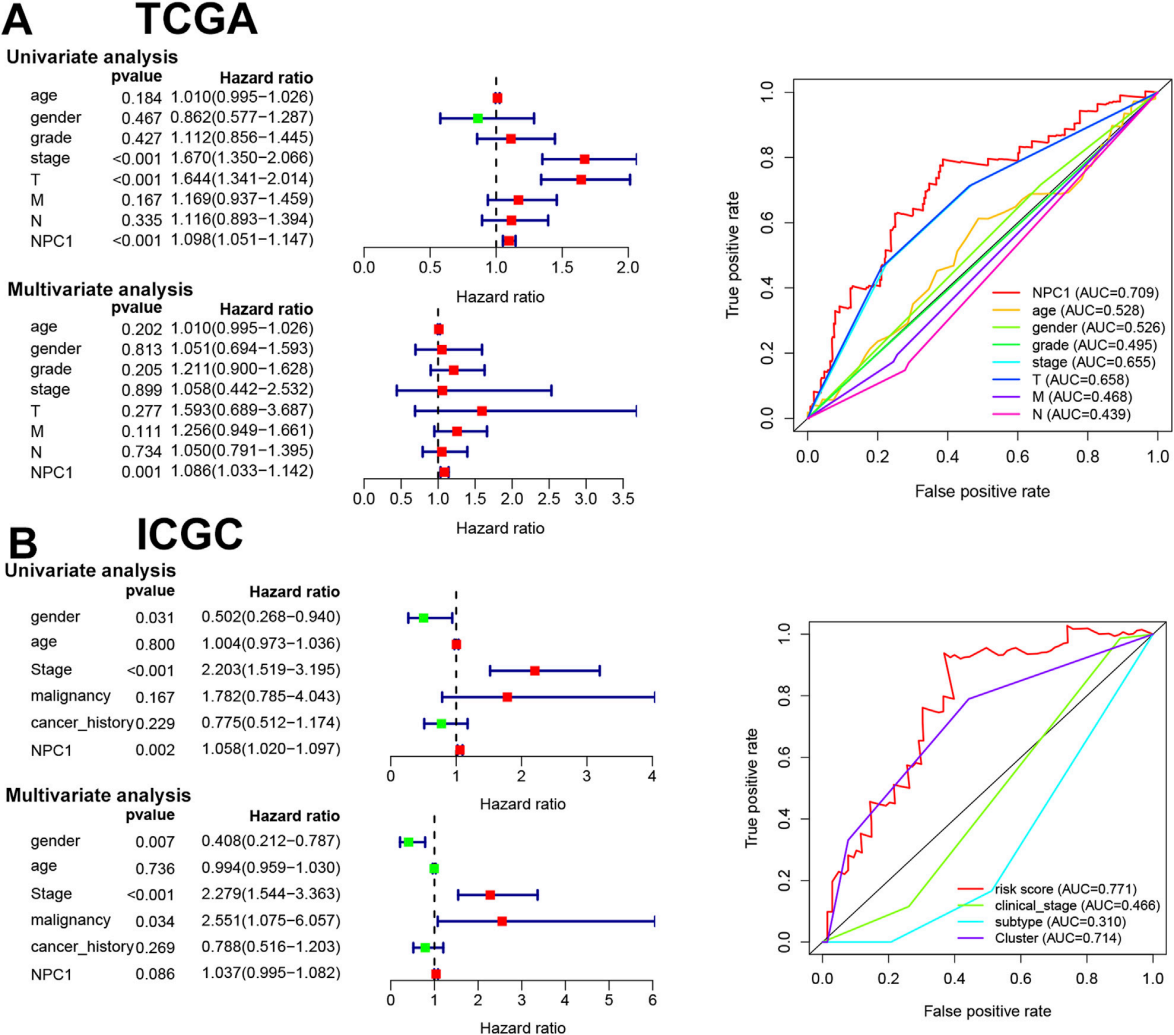
Prognostic value of NPC1 in HCC. The results of univariate and multivariate COX regression analyses and the ROC curve for NPC1 in relation to clinical characteristics of HCC, utilizing data from the TCGA **(A)** and ICGC databases **(B)**.

The objective of this study was to determine the prognostic significance of NPC1 in relation to clinical characteristics in LIHC patients. We conducted a comprehensive analysis using both univariate and multivariate Cox regression models to assess the association of NPC1 with OS in LIHC patients. Our analysis highlighted multiple factors that are significantly connected to OS in LIHC patients. These included cancer stage (HR = 1.67, P < 0.01), T stage (HR = 1.64, P < 0.01), and NPC1 expression levels (HR = 1.09, P < 0.01) in the TCGA database (Figure 4A). Similarly, the ICGC database analysis revealed gender (HR = 0.502, P = 0.031), stage (HR = 2.203, P < 0.01), and NPC1 (HR = 1.05, P < 0.01) as significant factors associated with OS in LIHC patients (Figure 4B). Upon further analysis using multivariate Cox regression, NPC1 (HR = 1.08, P < 0.01) was determined to be an independent prognostic factor in the TCGA database. This finding was corroborated by the ICGC database, where multivariate analysis identified gender (HR = 0.5, P < 0.01) and stage (HR = 1.058, P = 0.002) as independent prognostic factors for LIHC. To evaluate the predictive accuracy of NPC1 as a biomarker for HCC survival, we performed ROC (Receiver Operating Characteristic curve) analysis in both the ICGC and TCGA databases. The findings demonstrated that NPC1 was the most effective predictor of survival in LIHC patients across both datasets.

Then, we established NPC1 as an independent prognostic factor in LIHC, highlighting its potential utility as a predictive biomarker for patient survival. The consistent association of NPC1 with survival outcomes in multiple cohorts underscores its crucial role of NPC1 in HCC biology and its possible impact on clinical management strategies.

### 3.5 Analysis of NPC1 Co-expression networks in HCC

To uncover the genetic interactome associated with NPC1 and its functional implications in HCC, we employed the LinkedOmics database to identify genes that exhibit co-expression patterns with NPC1. This approach is predicated on the notion that gene co-expression networks can reveal shared genetic risk factors and functional linkages.

**FIGURE 5:**
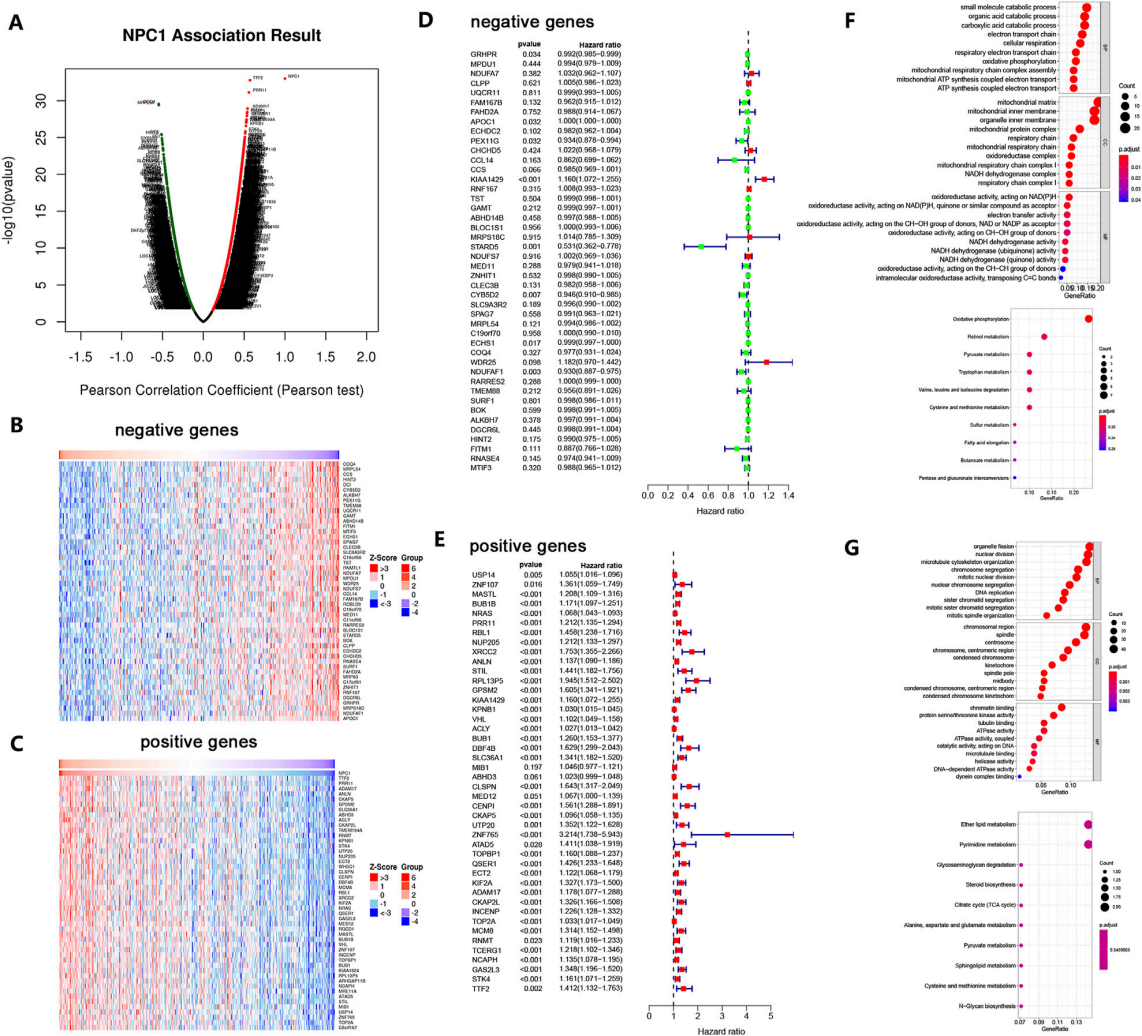
NPC1 Co-expression Network in HCC. The global co-expression analysis of NPC1 in the LIHC cohort as identified by Pearson’s correlation test **(A)**. Heat maps represent the top 50 genes that are negatively **(B)** and positively **(C)** correlated with NPC1 in LIHC, with red indicating positive correlation and blue indicating negative correlation. Survival maps for the top 50 genes negatively **(D)** and positively **(E)** correlated with NPC1 in LIHC are also provided. The figure concludes with significantly enriched GO annotations and KEGG pathways associated with NPC1 in the LIHC cohort, including GO enrichment and KEGG enrichment analyses for the top 50 negatively **(F)** and positively **(G)** correlated genes of NPC1.

The analysis revealed a cohort of genes that were significantly correlated with NPC1 expression in Hepatocellular Carcinoma. As depicted in Figure 5A, genes shown as dark red dots are strongly positively correlated with NPC1, while dark blue dots indicate a significant negative correlation, involving a total of 2,179 genes.

Using a heat map, the top 50 genes with significant correlations were identified, showing both negative and positive associations with NPC1 (Figures 5B,C). Notably, 47 of the top 50 genes with positive correlation were high-risk (Figure 5E), while eight of the top 50 genes with negative correlation were low-risk (Figure 5D).

To probe the possible biological functions of NPC1 in HCC, we selected the 50 genes with the strongest negative correlations for functional enrichment analysis. Gene Ontology (GO) and biological process (BP) analysis revealed significant enrichment in carboxylic acid catabolic process, organic acid catabolic process, and small molecule catabolic process. Cellular component (CC) enrichment analysis indicated that mitochondrial matrix, organelle inner membrane, and mitochondrial inner membrane were significantly enriched categories. Molecular function (MF) analysis indicated enrichment in oxidoreductase activity, specifically those acting on NAD(P)H, electron transfer activity, and quinone or similar compound as acceptor. KEGG enrichment analysis highlighted oxidative phosphorylation as a remarkably enriched pathway (Figure 5F).

Similarly, a functional enrichment analysis was conducted on the 50 genes with the highest positive correlation. GO-BP analysis indicated significant enrichment in organelle fission, nuclear division, and microtubule cytoskeleton organization. CC enrichment analysis revealed chromosomal region spindle and centrosome as significantly enriched categories. MF analysis showed enrichment in chromatin binding, tubulin binding, and protein serine/threonine kinase activity. KEGG enrichment analysis revealed remarkable enrichment in ether lipid metabolism and pyrimidine metabolism (Figure 5G).

These findings suggest that NPC1 could be crucial for DNA replication and other cellular processes, as indicated by the enrichment in pathways and functions related to cell division and metabolism. The co-expression network analysis not only underscores the multifaceted role of NPC1 in HCC but also provides a foundation for further investigation into its mechanistic involvement in tumorigenesis and potential as a therapeutic target.

## 4 Discussion

Hepatocellular carcinoma is a pervasive malignancy within the digestive system, characterized by rapid proliferation and early metastasis, and it ranks second in causing cancer-related mortality in China (Omata et al., 2017; Xie et al., 2020). Projections from the Global Cancer Observatory suggest that by 2025, approximately 431,000 new cases of primary liver cancer will be diagnosed in China, with HCC accounting for 75%–85% of these cases (Xie et al., 2023; Sung et al., 2021). The asymptomatic nature of HCC in its early stages often results in a diagnosis at an advanced stage, emphasizing the critical need for early diagnosis to improve treatment outcomes and survival rates (Feng et al., 2021; Sarbarzeh et al., 2020).

Cholesterol homeostasis is a pivotal factor in carcinogenesis, and NPC1, as a key regulator of extracellular cholesterol metabolism, is essential for the transport of free cholesterol from the lysosome to the cytoplasm (Riscal et al., 2019; Pfeffer, 2019; Davies et al., 2000). Our findings, along with those from previous studies, targeting NPC1 might be beneficial in cancer treatment owing to its role in cholesterol handling (Xu et al., 2020).

Our data indeed reveal marked heterogeneity in NPC1 expression across cancer types, with pronounced upregulation in pancreatic, liver, and other malignancies (Figure 1A), while showing reduced expression in six cancer types (Figure 1B). This paradoxical expression pattern can be mechanistically explained by three key factors: 1) Metabolic microenvironment dependency - NPC1’s cholesterol transport function is particularly critical in cancers with high lipid metabolic demands (e.g., pancreatic adenocarcinoma), where it modulates immunosuppressive tumor microenvironments by sequestering cholesterol from CD8^+^ T cells (Zi et al., 2025). Conversely, in cancers like thyroid or testicular tumors, cholesterol metabolism may be less dominant, leading to NPC1 downregulation. 2) Signaling pathway divergence - In hepatocellular carcinoma, NPC1 promotes tumor progression through TGF-β pathway stabilization independent of cholesterol transport, whereas in pancreatic cancer, its role is primarily immunomodulatory via β-catenin-LXR axis (Li et al., 2025). 3) Epigenetic regulation - DNA methylation patterns inversely correlate with NPC1 expression in Pancreatic adenocarcinoma (PAAD), suggesting tissue-specific epigenetic control mechanisms that may override oncogenic drivers in certain cancer types (Zi et al., 2025). These findings align with recent studies showing NPC1’s pleiotropic roles: its cholesterol-related functions dominate in immunologically “cold” tumors like pancreas, while non-metabolic roles (e.g., TGFBR1 stabilization) prevail in liver tumors (Li et al., 2025; Liu et al., 2024).

Further investigation into NPC1 expression in HCC through various databases confirmed higher mRNA and protein levels in cancerous tissues compared to normal tissues, suggesting NPC1 as a potential HCC biomarker. Consistent with our results, Xu et al. reported elevated NPC1 mRNA and protein levels in HCC cell lines and tissue samples compared to normal liver cell lines (Xu et al., 2024). Similar increases in NPC1 expression have been observed in clear cell renal cell carcinoma (ccRCC) (Fazliyeva et al., 2024) and gastric cancer (GC) (Tang et al., 2024), where high NPC1 expression correlated with an unfavorable prognosis. We used the Kaplan-Meier Plotter to reveal a strong positive correlation between high NPC1 expression and poor OS, RFS, and DFS in liver cancer. Additionally, the NPC1 expression in relation to various clinical indicators revealed statistically significant differences, and ROC analysis confirmed NPC1 as a reliable predictor of survival in HCC patients. These findings collectively suggest that NPC1 may act as an oncogenic driver, with high expression levels being closely associated with a poorer prognosis in HCC patients.

NPC1’s role in the biological mechanisms of cancer development is further supported by our GO and KEGG analyses, which indicated that the top 50 significant genes positively correlated with NPC1 are involved in functions critical to DNA replication. Studies have shown that NPC1 inhibition in triple-negative breast cancer reduces cellular proliferation and alters mitochondrial activity, among other effects (O'Neill et al., 2022). Du et al. also found that NPC1 depletion in multiple cancer cell lines inhibited both cell proliferation and migration (Du et al., 2015). These findings collectively suggest that targeting NPC1 may have therapeutic potential in cancer treatment.

Our study was significantly limited by the absence of direct experimental data demonstrating the impact of NPC1 modulation on HCC or the behavior of HCC cells. Although our findings indicated a strong association between NPC1 expression and HCC progression, highlighting its potential as a biomarker, they did not conclusively prove NPC1 as a therapeutic target. Future research will focus on determining the causal role of NPC1 in promoting HCC malignancy and assessing the therapeutic potential of its inhibition, thus offering essential functional proof to confirm NPC1 as a therapeutic target.

In summary, our findings converge to suggest that NPC1 overexpression is a distinguishing feature of HCC and is linked to adverse prognosis, highlighting its potential utility as a predictive biomarker. Moreover, the inhibitory effects of NPC1 downregulation on key oncogenic properties of HCC cells imply that NPC1 could be a viable therapeutic target. These insights collectively advocate for further investigation into the mechanistic role of NPC1 in HCC pathogenesis and the development of targeted NPC1 therapies. Future studies should focus on elucidating the precise molecular mechanisms through which NPC1 influences HCC progression and on assessing the efficacy of NPC1-targeted interventions in preclinical and clinical settings.

## 5 Conclusion

The comprehensive analysis presented herein has delineated a robust association between elevated NPC1 expression and hepatocellular carcinoma (HCC) tissues, in contrast to normal liver tissues. Our results not only substantiate the correlation between NPC1 expression levels and clinical outcomes in HCC but also position NPC1 as a potential independent prognostic biomarker for this aggressive cancer type. The significance of NPC1 in HCC is further underscored by the observed effects of NPC1 gene suppression on the proliferation and invasive capabilities of HCC cells.

## Data Availability

The datasets presented in this study can be found in online repositories. The names of the repository/repositories and accession number(s) can be found in the article/Supplementary Material.
