# Supplementary material for: A comprehensive analysis identifies and validates NPC1 as a potential biomarker for prognosis in HCC
Source: Front Genet. 2025 Aug 13;16:1588583. doi: 10.3389/fgene.2025.1588583 (PMC12380838; doi:10.3389/fgene.2025.1588583)
Supplement: Supplementary file 1 [file Image1.pdf]

Supplementary Material

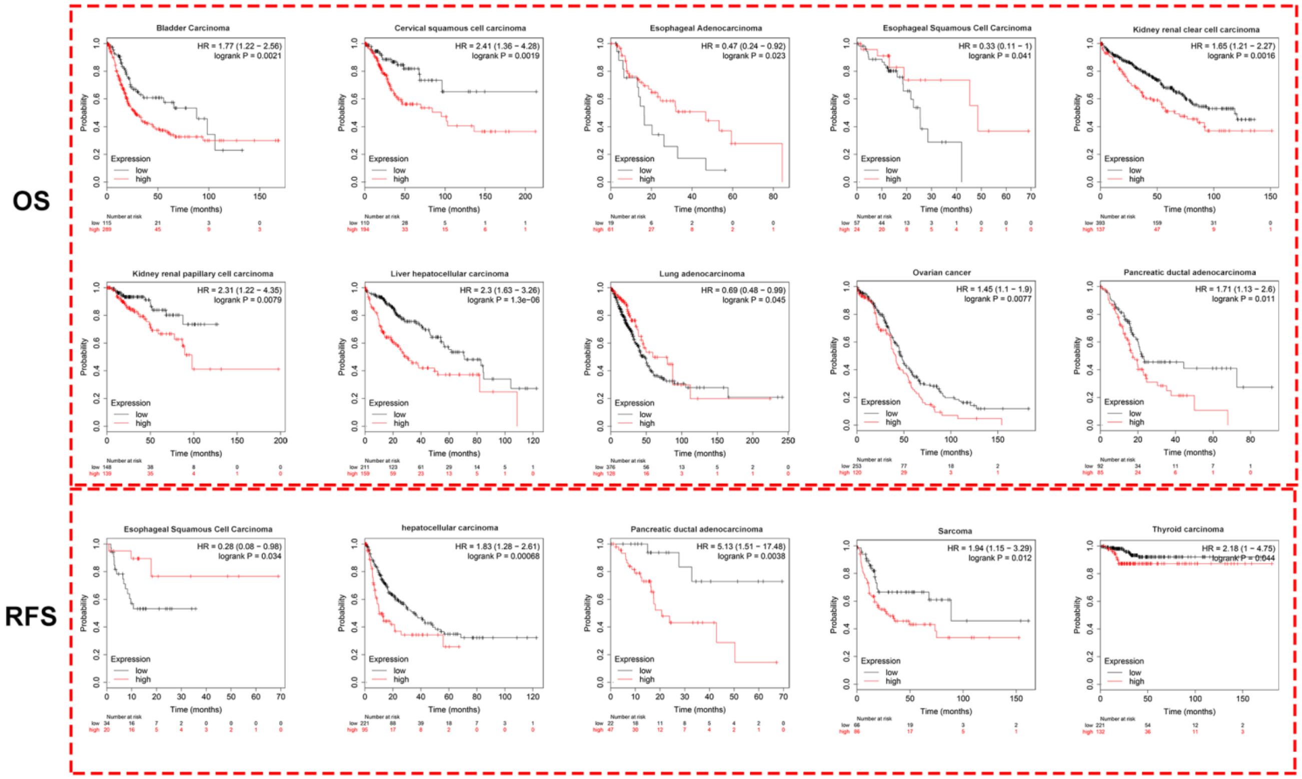

Supplementary Figure 1. NPC1 expression and OS and RFS relevance in multi- tumours.
